# Supplementary material for: First validity testing of GluciQuizz, a French self-questionnaire evaluating carb-counting for patients with type 1 diabetes
Source: PLoS One. 2025 Feb 25;20(2):e0318746. doi: 10.1371/journal.pone.0318746 (PMC11856297; doi:10.1371/journal.pone.0318746)
Supplement: S1 Table — The table shows the different steps in the validation process of the questionnaire (*step number as described in Table 1 of the manuscript). ACQ US, AdultCarbQuiz original version; ACQ French, AdultCarbQuiz translated into French; ACQ French adapted, questionnaire after cross-cultural adaptation for French people; Clarity, Consistency, Relevance and Sufficiency, expert notes for each item; % of correct answers of 190 participants living with T1D; Removed items, item was removed when more than 95% of patients had the same score for the same modality; Cronbach’s α, inconsistent items where removal resulted in a slight increase of global Cronbach’s α coefficient are presented in bold. * For sufficiency, the rating was applied to each section as a whole, not to individual items. (DOCX) [file pone.0318746.s001.docx]

S1 Table. Domain 1 of AdultCarbQuiz and GluciQuizz: carbo­hydrate food recognition

| Steps* | Step 1 | Step 2 | Step 3 | | | | Step 5 | Step 6 | Step 7 |
| --- | --- | --- | --- | --- | --- | --- | --- | --- | --- |
| AdultCarbQuiz | French translation | *Cross-cultural adaptation* | Face validity | | | | Internal consistency | Simplification | Evaluation of discrimination |
|  |  |  | Clarity | Consis  tency | Relev  ance | Suffic  iency | % correct responses | Removed  Items | Cronbach’s α |
| Bread | Pain |  | 3.85 | 3.62 | 3.92 | 3.62* | 98.9 | X |  |
| Breakfast Sausages | Saucisses du petit déjeuner | Saucisses de Strasbourg | 3.62 | 3.08 | 3.46 |  | 67.4 |  | 0.779 |
| Baked potato | Pomme de terre au four |  | 3.69 | 3.62 | 3.77 |  | 97.9 | X |  |
| Regular Maple syrup | Sirop d’érable ordinaire | Cabillaud (poisson blanc) | 3.62 | 2.85 | 3.31 |  | 94.7 |  | 0.780 |
| American cheese | Fromage américain | Gruyère | 3.69 | 3.15 | 3.46 |  | 66.8 |  | 0.776 |
| Low-fat milk | Lait demi-écrémé |  | 3.69 | 3.08 | 3.46 |  | 72.6 |  | 0.783 |
| Apple juice | Jus de pommes |  | 3.85 | 3.62 | 3.85 |  | 96.3 | X |  |
| Soda pop (not diet) | Soda |  | 3.85 | 3.62 | 3.85 |  | 95.8 |  | 0.780 |
| Cooked dried beans (navy beans, lentils) | Grains secs cuisinés (haricot blanc, lentilles) | Légumes secs (lentilles, flageolets, haricots rouges, …) | 3.77 | 3.46 | 3.77 |  | 86.3 |  | 0.778 |
| Apple | Pomme |  | 3.85 | 3.62 | 3.85 |  | 93.7 |  | 0.780 |
| Sugar | Sucre | Haricots verts | 3.62 | 2.92 | 3.38 |  | 63.2 |  | **0.794** |
| Butter | Beurre |  | 3.69 | 2.92 | 3.31 |  | 75.8 |  | 0.772 |
| Cooked rice | Riz cuit |  | 3.85 | 3.62 | 3.85 |  | 96.8 | X |  |
| Plain grilled chicken | Poulet grillé |  | 3.62 | 2.77 | 3.31 |  | 86.3 |  | 0.772 |
| Blackberry jam | Confiture de mûre | Confiture de fraises | 3.85 | 3.54 | 3.85 |  | 97.4 | X |  |
| Cooked spaghetti noodles (no sauce) | Spaghetti cuites natures |  | 3.85 | 3.62 | 3.85 |  | 95.8 |  | 0.780 |
| Canned spaghetti sauce (tomato) | Sauce à spaghetti en conserve | Sauce tomate (style “ketchup”) | 3.77 | 3.54 | 3.77 |  | 90.0 |  | 0.781 |
| Hamburger patty | Steak haché cuit |  | 3.62 | 2.77 | 3.31 |  | 83.7 |  | 0.771 |
| Honey | Miel |  | 3.85 | 3.62 | 3.85 |  | 96.3 | X |  |

The table shows the different steps in the validation process of the questionnaire (*step number as described in Table 1 of the manuscript)

ACQ US, AdultCarbQuiz original version; ACQ French, AdultCarbQuiz translated into French; ACQ French adapted, questionnaire after cross-cultural adaptation for French people; Clarity, Consistency, Relevance and Sufficiency, expert notes for each item; % of correct answers of 190 participants living with T1D; Removed items, item was removed when more than 95% of patients had the same score for the same modality; Cronbach’s α, inconsistent items where removal resulted in a slight increase of global Cronbach’s α coefficient are presented in **bold**. * For sufficiency, the rating was applied to each section as a whole, not to individual items.
