# Supplementary material for: First validity testing of GluciQuizz, a French self-questionnaire evaluating carb-counting for patients with type 1 diabetes
Source: PLoS One. 2025 Feb 25;20(2):e0318746. doi: 10.1371/journal.pone.0318746 (PMC11856297; doi:10.1371/journal.pone.0318746)
Supplement: S2 Table — ACQ US, AdultCarbQuiz original version; ACQ French, AdultCarbQuiz translated into French; ACQ French adapted, questionnaire after cross-cultural adaptation for French people; Clarity, Consistency, Relevance and Sufficiency, expert notes for each item; % of correct answers of 190 participants living with T1D; Removed items, item was removed when more than 95% of patients had the same score for the same modality; Cronbach’s α, inconsistent items where removal resulted in a slight increase of global Cronbach’s α coefficient are presented in bold. * For sufficiency, the rating was applied to each section as a whole, not to individual items. (DOCX) [file pone.0318746.s002.docx]

S2 Table. Domain 2 of AdultCarbQuiz and GluciQuizz: carbohydrate food content.

| ACQ US | ACQ French | ACQ French adapted | Clarity | Consis  tency | Relev  ance | Suffic  iency | % correct responses of all participants  n=190 | Removed  Items | Cronbach’s α |
| --- | --- | --- | --- | --- | --- | --- | --- | --- | --- |
| 1 cup milk | 1 verre de lait (200mL) |  | 3.77 | 3.46 | 3.46 | 3.54* | 56.3 |  | 0.772 |
| 1 cup pasta | 1 assiette de pâtes cuites (200g) |  | 3.85 | 3.85 | 3.77 |  | 20.0 |  | 0.783 |
| 1 cup cooked rice | 1 assiette de riz cuit (200g) |  | 3.85 | 3.77 | 3.69 |  | 38.9 |  | 0.775 |
| 1 cup 100% juice | 1 verre de jus d’orange (200mL) |  | 3.77 | 3.77 | 3.77 |  | 59.5 |  | 0.771 |
| 1 cup hot cereal | 1/2 bol céréales sucrées (45g) |  | 3.69 | 3.77 | 3.69 |  | 16.8 |  | 0.781 |
| 1 cup cooked dried beans | 1 assiette de flageolets (200g) |  | 3.85 | 3.69 | 3.62 |  | 28.4 |  | 0.783 |
| 1 cup mashed potatoes | 1 assiette de purée (200g) |  | 3.85 | 3.77 | 3.85 |  | 37.4 |  | 0.782 |

ACQ US, AdultCarbQuiz original version; ACQ French, AdultCarbQuiz translated into French; ACQ French adapted, questionnaire after cross-cultural adaptation for French people; Clarity, Consistency, Relevance and Sufficiency, expert notes for each item; % of correct answers of 190 participants living with T1D; Removed items, item was removed when more than 95% of patients had the same score for the same modality; Cronbach’s α, inconsistent items where removal resulted in a slight increase of global Cronbach’s α coefficient are presented in **bold**. * For sufficiency, the rating was applied to each section as a whole, not to individual items.
