# Supplementary material for: First validity testing of GluciQuizz, a French self-questionnaire evaluating carb-counting for patients with type 1 diabetes
Source: PLoS One. 2025 Feb 25;20(2):e0318746. doi: 10.1371/journal.pone.0318746 (PMC11856297; doi:10.1371/journal.pone.0318746)
Supplement: S3 Table — ACQ US, AdultCarbQuiz original version; ACQ French, AdultCarbQuiz translated into French; ACQ French adapted, questionnaire after cross-cultural adaptation for French people; Clarity, Consistency, Relevance and Sufficiency, expert notes for each item; % of correct answers of 190 participants living with T1D; Removed items, item was removed when more than 95% of patients had the same score for the same modality; Cronbach’s α, inconsistent items which removal resulted in a slight increase of global Cronbach’s α coefficient are presented in bold. * For sufficiency, the rating was applied to each section as a whole, not to individual items. (DOCX) [file pone.0318746.s003.docx]

S3 Table. Domain 3 of GluciQuizz: nutrition label reading.

| ACQ US | ACQ French | ACQ French adapted | Clarity | Consis  tency | Relev  ance | Suffic  iency | % correct responses | Suppre  items | Cronbach’s α |
| --- | --- | --- | --- | --- | --- | --- | --- | --- | --- |
| Look at the Nutrition Facts label. what is the serving size? *1 cup 2 cups 4 cups Unsure* |  | A quelle partie de l’étiquette devez-vous vous référer pour un bol de céréales (1 portion) afin de connaître l’apport glucidique ?  *Je ne sais pas Partie supérieure*  *Partie inférieure Les deux* | 3.62 | 3.85 | 3.69 | 3.38* | 69.5 |  | 0.779 |
| For one serving, how much carbohydrate would you eat in grams?  *228g 5g 31g Unsure* |  | Pour une portion, combien de glucides mangerez-vous, en grammes ?  *Je ne sais pas 62g 11g 28g* | 3.54 | 3.85 | 3.77 |  | 82.6 |  | 0.777 |
| If you ate the whole package, how many cups would you eat? *1 cup 2 cups 4 cups Unsure* |  | Si vous mangez le paquet entier, combien de portions mangez-vous ?  *Je ne sais pas 10 16*  *22* | 3.38 | 3.85 | 3.69 |  | 77.4 |  | 0.773 |
| If you ate the whole package, how much carbohydrate would you eat in grams?  *456g 10g 62g Unsure* |  | Est-ce que ces céréales contiennent des lipides ?  *Oui Non* | 3.46 | 3.69 | 3.31 |  | 78.4 |  | 0.784 |


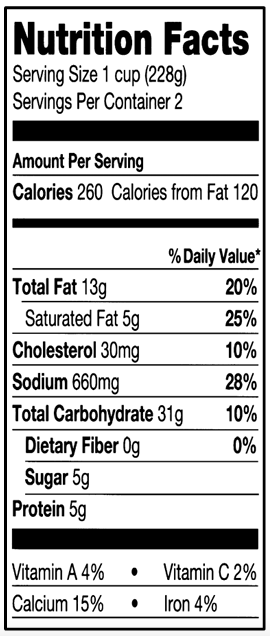


ACQ US, AdultCarbQuiz original version; ACQ French, AdultCarbQuiz translated into French; ACQ French adapted, questionnaire after cross-cultural adaptation for French people; Clarity, Consistency, Relevance and Sufficiency, expert notes for each item; % of correct answers of 190 participants living with T1D; Removed items, item was removed when more than 95% of patients had the same score for the same modality; Cronbach’s α, inconsistent items which removal resulted in a slight increase of global Cronbach’s α coefficient are presented in **bold**. * For sufficiency, the rating was applied to each section as a whole, not to individual items.
