# Supplementary material for: First validity testing of GluciQuizz, a French self-questionnaire evaluating carb-counting for patients with type 1 diabetes
Source: PLoS One. 2025 Feb 25;20(2):e0318746. doi: 10.1371/journal.pone.0318746 (PMC11856297; doi:10.1371/journal.pone.0318746)
Supplement: S4 Table — ACQ US, AdultCarbQuiz original version; ACQ French, AdultCarbQuiz translated into French; ACQ French adapted, questionnaire after cross-cultural adaptation for French people; Clarity, Consistency, Relevance and Sufficiency, expert notes for each item; % of correct answers of 190 participants living with T1D; Removed items, item was removed when more than 95% of patients had the same score for the same modality; Cronbach’s α, inconsistent items which removal resulted in a slight increase of global Cronbach’s α coefficient are presented in bold. * For sufficiency, the rating was applied to each section as a whole, not to individual items. (DOCX) [file pone.0318746.s004.docx]

S4 Table. Domain 4 of GluciQuizz (corresponding to Domains 4 and 5 of AdultCarbQuiz): gly­cemic targets. hypoglycemia prevention and treatment.

| ACQ US | ACQ French | ACQ French adapted | Clarity | Consis  tency | Relev  ance | Suffic  iency | % correct responses | Removed  items | Cronbach’s α |
| --- | --- | --- | --- | --- | --- | --- | --- | --- | --- |
| Which will make your blood sugar go up higher: eating 3 carbs or 5 carbs?  *3 5 Unsure* | Un taux de glucose préprandiale (juste avant le repas) satisfaisant est de :  *Je ne sais pas 60mg/dL 110mg/dL 180mg/dL* |  | 3.85 | 3.85 | 3.85 | 3.78* | 95.3 | X |  |
| A good blood sugar reading just before a meal would be?  *60 110 180 Unsure* | Un taux de glucose préprandiale (juste avant le repas) satisfaisant est de :  *Je ne sais pas 60mg/dL 110mg/dL 180mg/dL* |  | 3.85 | 3.85 | 3.77 |  | 92.1 |  | 0.783 |
| A good blood sugar reading 2 hours after a meal would be?  *60 140 220 Unsure* | Un taux de glucose post-prandiale (2 heures après le repas) satisfaisant est de  *Je ne sais pas 60mg/dL 140mg/dL 220mg/dL* |  | 3.77 | 3.77 | 3.77 |  | 93.2 |  | 0.782 |
| One “carb choice” is equal to how many grams of carbohydrates?  *15 5 25 Unsure* | Combien de grammes de glucides sont contenus dans un morceau de sucre standard ?  *Je ne sais pas 5g 15g 25g* |  | 3.85 | 3.85 | 3.85 |  | 78.4 |  | 0.776 |
| One carb choice will bring up your blood sugar by how many points? *500 10 50 Unsure* | De quel ordre de grandeur augmente le taux de glucose après la prise d’un morceau de sucre ?  *Je ne sais pas 500mg/dL 10mg/dL 50mg/dL* |  | 3.77 | 3.77 | 3.77 |  | 32.6 |  | **0.787** |
| Which of these carb foods will bring up your blood sugar the fastest?  *Glucose tablets Candy bar Bread Unsure* | Quel aliment augmentera le plus rapidement la glycémie ?  *Je ne sais pas Morceau de sucre Barre chocolatée Pain complet* |  | 3.85 | 3.85 | 3.85 |  | 93.7 |  | 0.783 |
| You are going to mow the grass, which takes about 30 minutes of solid work. By how many points do you expect your blood sugar to go down?  *25 50 150 Unsure* | Vous êtes sur le point de tondre la pelouse, ce qui correspond à environ 30 min d’effort physique vigoureux. De quel ordre de grandeur pensez-vous diminuer votre taux de glucose ?  *Je ne sais pas 10mg/dL 50mg/dL 150mg/dL* |  | 3.46 | 3.77 | 3.69 |  | 54.7 |  | **0.786** |
| You just walked fast for one hour and you start to feel shaky and nervous. Before you started walking, your blood sugar was 160. What is it now?  *210 160 60 Unsure* | Vous venez juste de faire une marche rapide pendant une heure et vous commencez à trembler et à vous sentir faible. Avant de débuter la marche, votre taux de glucose était de 160mg/dL. A combien est-il maintenant ?  *Je ne sais pas 210mg/dL 160mg/dL 60mg/dL* |  | 3.62 | 3.69 | 3.61 | 3.61 | 94.2 |  | 0.782 |
| You get a low blood sugar of 50. How many hard candies should you eat to bring up your blood sugar 50 points?  *1 5 10 Unsure* | Votre taux de glucose est bas à 50 mg/dL. Combien de morceaux de sucre devez-vous manger pour augmenter votre taux de glucose de 50mg/dL ?  *Je ne sais pas 1 5 10* |  | 3.92 | 3.92 | 3.92 | 3.69 | 40.5 |  | **0.794** |

ACQ US, AdultCarbQuiz original version; ACQ French, AdultCarbQuiz translated into French; ACQ French adapted, questionnaire after cross-cultural adaptation for French people; Clarity, Consistency, Relevance and Sufficiency, expert notes for each item; % of correct answers of 190 participants living with T1D; Removed items, item was removed when more than 95% of patients had the same score for the same modality; Cronbach’s α, inconsistent items which removal resulted in a slight increase of global Cronbach’s α coefficient are presented in **bold**. * For sufficiency, the rating was applied to each section as a whole, not to individual items.
