# Supplementary material for: First validity testing of GluciQuizz, a French self-questionnaire evaluating carb-counting for patients with type 1 diabetes
Source: PLoS One. 2025 Feb 25;20(2):e0318746. doi: 10.1371/journal.pone.0318746 (PMC11856297; doi:10.1371/journal.pone.0318746)
Supplement: S5 Table — ACQ US, AdultCarbQuiz original version; ACQ French, AdultCarbQuiz translated into French; ACQ French adapted, questionnaire after cross-cultural adaptation for French people; Clarity, Consistency, Relevance and Sufficiency, expert notes for each item; % of correct answers of 190 participants living with T1D; Removed items, item was removed when more than 95% of patients had the same score for the same modality; Cronbach’s α, inconsistent items which removal resulted in a slight increase of global Cronbach’s α coefficient are presented in bold. * For sufficiency, the rating was applied to each section as a whole, not to individual items. (DOCX) [file pone.0318746.s005.docx]

S5 Table. Domain 5 of GluciQuizz (corresponding to Domain 6 of AdultCarbQuiz): carbohydrate content of meals.

| ACQ US | ACQ French | ACQ French adapted | Clarity | Consis  tency | Relev  ance | Suffic  iency | % correct responses | Removeditems | Cronbach’s α |
| --- | --- | --- | --- | --- | --- | --- | --- | --- | --- |
| Breakfast: 2 eggs and 2 sausage patties, 2 cups black coffee with sweetener. | Petit déjeuner : 2 œufs, 2 saucisses, 2 cafés noir avec sucrette. | Petit déjeuner : 1 pain au chocolat de boulangerie, 1 verre de jus d’orange, 1 café noir avec 2 sucrettes | 3.85 | 3.69 | 3.84 | 3.77* | 47.9 |  | 0.773 |
| Lunch: 1 sandwich, 1 dill pickle and 1 20-ounce bottle of fruit juice. | Déjeuner : 1 sandwich, 1 grand cornichon, 600ml de jus de fruit | Déjeuner : 1 grand sandwich poulet crudité, 1 part de flan pâtissier, 1 soda « zéro » | 3.69 | 3.62 | 3.76 |  | 36.3 |  | 0.774 |
| Snack: 1 large banana | 1 grande banane | 1 barre chocolatée | 3.77 | 3.54 | 3.84 |  | 23.7 |  | **0.787** |
| Supper: Half (4 pieces) of a large pizza, green salad and iced tea with sweetener | Diner: moitié (4 pièces) d’un pizza grand taille, salade verte, thé glacé avec sucrette | Diner : 1 pizza taille « restaurant », salade verte, 1 soda | 3.54 | 3.69 | 3.84 |  | 56.8 |  | 0.784 |

ACQ US, AdultCarbQuiz original version; ACQ French, AdultCarbQuiz translated into French; ACQ French adapted, questionnaire after cross-cultural adaptation for French people; Clarity, Consistency, Relevance and Sufficiency, expert notes for each item; % of correct answers of 190 participants living with T1D; Removed items, item was removed when more than 95% of patients had the same score for the same modality; Cronbach’s α, inconsistent items which removal resulted in a slight increase of global Cronbach’s α coefficient are presented in **bold**. * For sufficiency, the rating was applied to each section as a whole, not to individual items.
