# Supplementary figures and images for: First validity testing of GluciQuizz, a French self-questionnaire evaluating carb-counting for patients with type 1 diabetes
Source: PLoS One. 2025 Feb 25;20(2):e0318746. doi: 10.1371/journal.pone.0318746 (PMC11856297; doi:10.1371/journal.pone.0318746)

S1 File. GluciQuizz.
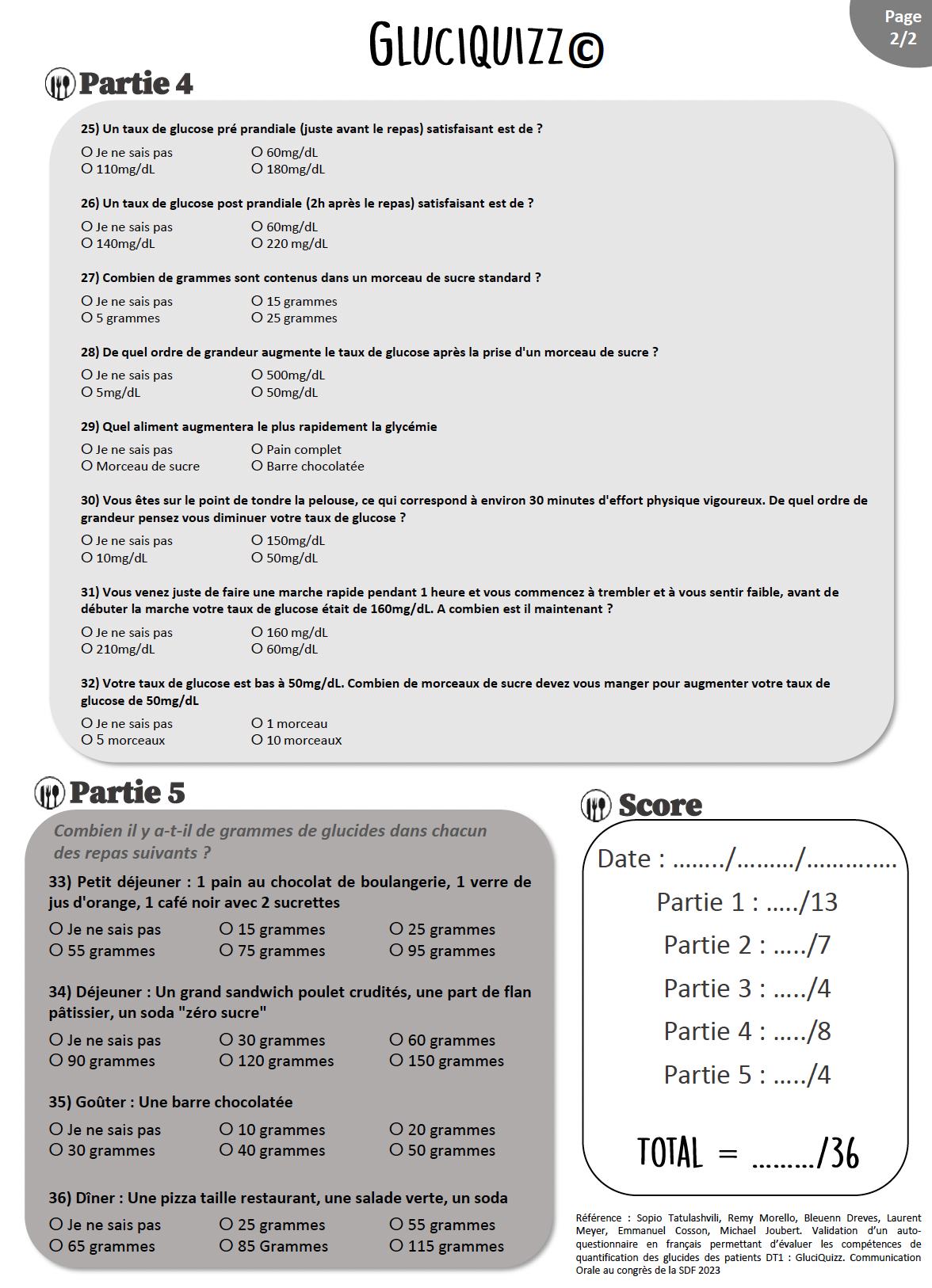

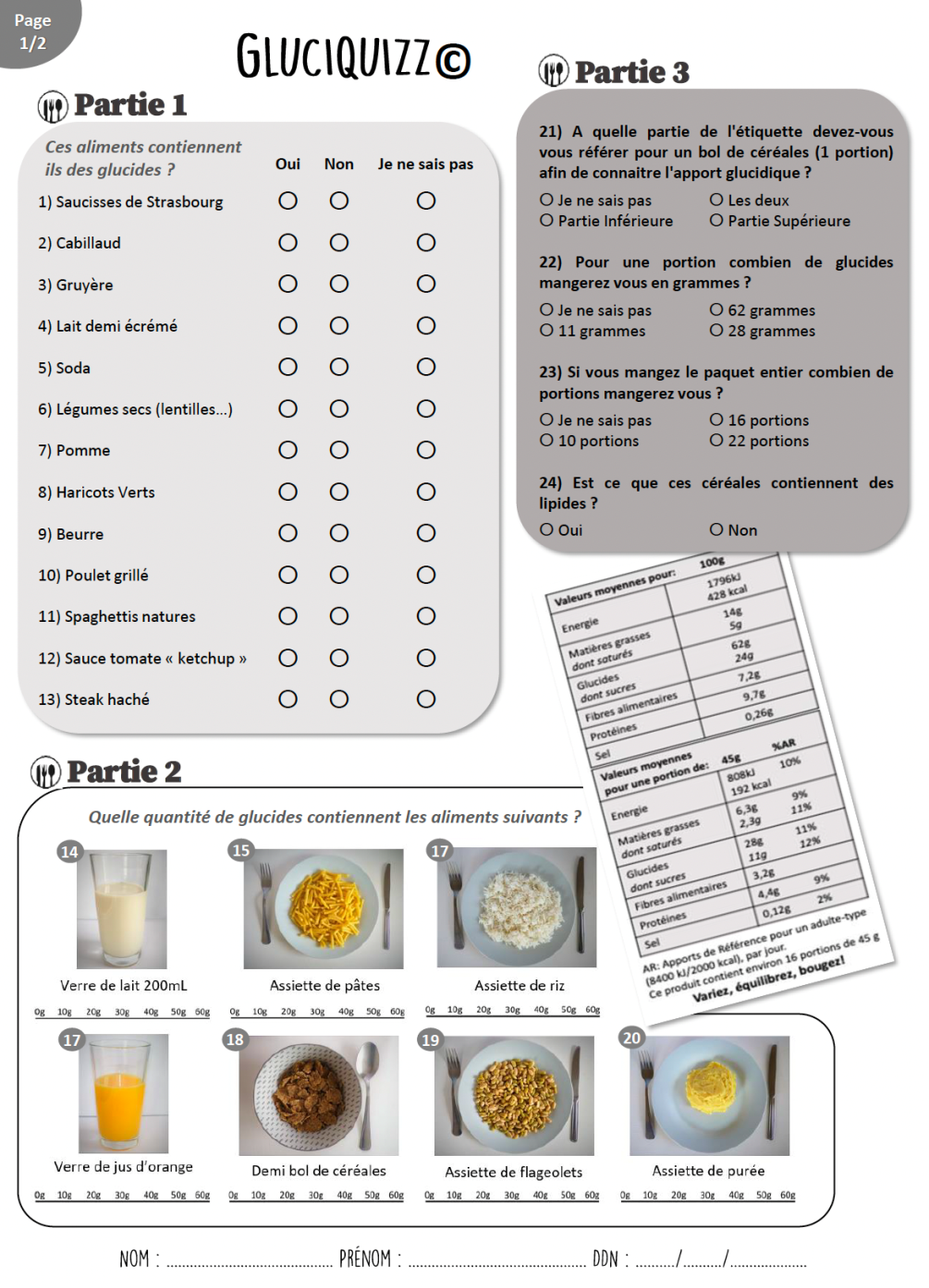
 Full questionnaire.

Supplement: S1 File — Full questionnaire. (DOCX) [file pone.0318746.s006.docx]
