# Supplementary material for: First validity testing of GluciQuizz, a French self-questionnaire evaluating carb-counting for patients with type 1 diabetes
Source: PLoS One. 2025 Feb 25;20(2):e0318746. doi: 10.1371/journal.pone.0318746 (PMC11856297; doi:10.1371/journal.pone.0318746)
Supplement: Attachment — Submitted filename: Response to reviewers.docx [file pone.0318746.s007.docx]

5. Review Comments to the Author

Please use the space provided to explain your answers to the questions above. You may also include additional comments for the author, including concerns about dual publication, research ethics, or publication ethics. (Please upload your review as an attachment if it exceeds 20,000 characters)

Reviewer #1: I reviewed the article untitled "First validity testing of GluciQuizz, a French self-questionnaire evaluating carb-counting for patients with type 1 diabetes" which addresses the need for a French questionnaire to assess carbohydrate counting skills in French adults with T1D.
The cultural adaptation of such a questionnaire is a thorough work that requires several stages and participants. Overall, the manuscript is well written, and each step is well described. Therefore, most of my comments are minor.
A small note, I was not able to access the supplementary material, therefore some of my comments might have been addressed if I had access to it.

Response: Thank you for your positive evaluation of our work.

1. In the introduction, line 60, the authors state that HCL allows optimal glycemic control at night and between meals. I would rephrase this to say that HCL provides better overall glycemic control (compared to other modes of insulin delivery), especially control at night and between meals.

Response: Thank you, we changed the sentence.

2. In the introduction, line 64, the authors refer to meals and carbohydrate quality. Do they mean meal composition and carbohydrate counting (according to the reference)?

Response: Indeed, we have replaced the word ‘quality’ by ‘meal composition and glycemic index’ in the manuscript.

3. In the methods (page 7, first paragraph), the cut-off to decide whether clarity/consistency/relevance... is acceptable is not defined a priori.

Answer: We have added the sentence to the manuscript: ‘responses rated at 3 and 4 were considered as satisfactory’.

4. In Table 1, last row, some text seems to be missing "Correlation between GluciQuizz" and?

Answer: thank you for this remark. We have completed the table 1 with the following sentence: ‘Correlation between GluciQuizz score and percentage of time in range (TIR)’.

5. On page 8, step 8, one could say that assessing post-meal TIR would have been even more sensitive. Just a comment.

Answer: We agree with your comment; however, unfortunately, we did not have information about precise meal times to assess post-meal TIR. We added this comment in the discussion.

6. On page 8, line 150, I do not clearly understand why there are only 5 domains in the French version versus 6 domains in the US version. Were two domains combined and why?

Answer: Indeed, domains 4 and 5 of the original questionnaire were combined, as they explore similar areas, such as glycemic targets and the use of carbohydrates in various situations like hypoglycemia and physical activity.

7. In Table 2, it would have been more readable to have the full domain title in the heading instead of the numbers. Same for AE/N/A. I think this could be done by changing the table to a landscape layout and putting the range on the same row as the mean, thus reducing the height of the rows.

Answer: We have made the changes to table 2 as suggested

8. In Table 3, it would have been nice to show the educational level of the patients who answered the questionnaire, since carbohydrate counting skills are associated with socioeconomic and education background. Especially since the authors found 9 items to be non-discriminatory due to a high rate of correct answers.

Answer: We agree with this comment; however, we unfortunately do not have information about the education level of participants. Another study, which is currently in progress, is addressing this question.

9. On page 13, line 187-188, sorry if this sounds silly as I am not a questionnaire expert, but would it make sense to calculate the Cronbach separately for each group of domains assessed? Or is only the overall Cronbach important?

Answer: It is indeed an excellent question that would make sense if the scale could be used only for one of its domains or another. This scale is interesting in a global approach, hence our choice to validate it in its entirety.

Comment: Thank you very much for your careful review and your constructive remarks.

Reviewer #2: Thank you for inviting me to review this manuscript “First validity testing of GluciQuizz, a French self-questionnaire evaluating carb-counting for patients with type 1 diabetes.
The article is presented in simple language and is easy to follow. The authors have done a comprehensive validity testing process including, translation, adaptation, back translation, testing face validity, testing with target group and experts for the content and reliability testing in a systematic way.
Reliability coefficients of 0.738-0.826 indicates high internal consistency. I believe predictive validity is not required as it is an adapted version for lanuage and minor cultural preferences.
I am not proficient in French and cannot comment for any language error in the Gluciquizz.

Comment: Thank you very much for your careful review and your constructive remarks.

Reviewer #3: Dear Authors,
This French-speaking self-administered GluciQuizz validation study questionnaire is essential to French patients. Studies show that carbohydrate counting is a valuable tool for better glucose control and reduced hypoglycemia, and it is crucial to evaluate carbohydrate knowledge and cross-cultural adaptation to help patients learn the carbohydrate count appropriately. The authors followed all the steps recommended for a validation study. However, the manuscript deserves writing revision due to some grammar, punctuation, and spelling mistakes.

Answer: Thank you very much for your positive evaluation of our work. We have taken this remark into account and made the necessary corrections to the manuscript.
